# Life Satisfaction of Rural-To-Urban Migrants: Exploring the Influence of Socio-Demographic and Urbanisation Features in China

**DOI:** 10.3389/ijph.2022.1604580

**Published:** 2022-06-22

**Authors:** Xiangjing Zhang, Wusi Zhou

**Affiliations:** ^1^ School of Public Administration, Hangzhou Normal University, Hangzhou, China; ^2^ Center for Population and Development Policy Studies, Fudan University, Shanghai, China

**Keywords:** urbanisation, life satisfaction, personal health, rural villager, community environment

## Abstract

**Objectives:** China has experienced an ongoing urbanisation that associated with spatial transformation and personal changes, which are expected to have direct or indirect impacts on migrants’ health and well-being. This study aims to investigate their life satisfaction and the significant influences.

**Method:** A quantitative research strategy was adopted, with the questionnaire survey towards 877 migrants across Hangzhou and Ningbo cities. Descriptive statistics showed migrants’ life satisfaction, socio-demographic characteristics and urbanisation features. Three logistic regression models examined key factors that influenced life satisfaction.

**Results:** Over one third of migrants were unsatisfied with their life. They mainly received education lower than high school and had an annual household income less than 60k. Comparatively, migrants, who worked in formal sectors, moved into resettlement communities and adapted to city life, tended to have higher levels of life satisfaction.

**Conclusion:** There should be an improvement in migrants’ life satisfaction. This could be achieved by building up their educational level and financial capability. Meanwhile, positive actions such as professional training are required to maximise their participation in formal sectors as well as community life.

## Introduction

Urbanisation is a socio-economic process that shifts the spatial contribution of population from rural to urban areas [1]. It has a positive impact on economic growth, human development and poverty reduction [2, 3]. However, the rapid urbanisation process has caused a range of problems, such as unsettled migrants, income inequity and land scarcity, leading to enormous challenges on sustainable development [4]. To address these challenges, it is crucial to promote urban sustainability [5]. According to Rasoolimanesh et al. [6], sustainable urbanisation is mainly dependent upon two factors: the sustainable city and sustainable urban development. A sustainable city is closely related to the city’s conditions, including resource use, environment protection, personal welfare and life satisfaction [7]. Sustainable urban development refers to a dynamic space in which economic, environmental and social ties are built up and well managed [8, 9]. In this regard, life satisfaction, as a core indicator of quality of life, plays a potential role in shaping urban development and further sustainable urbanisation.

Since the economic reforms in 1978, China has been experiencing an unprecedented urbanisation, resulting in the large-scale flow of rural-to-urban mitigation [10]. Until 2019, the level of urbanisation has reached 60.60%, with nearly 680 million of population leaving rural areas and moving to urban areas [11]. Some empirical studies have investigated various aspects of China’s urbanisation, such as its distinctive features [12], current implementation [13], performance outcomes [14] and policy interventions [15]. Some research experiences found that urbanisation was accompanied with some changes in migrants’ livelihood, including their home environment, occupations, behaviour and lifestyle [1, 16]. These changes are frequently linked to the levels of life satisfaction [17]. Accordingly, a branch of research examined the relationship between urbanisation and life satisfaction [18, 19], resulting in two distinct views. On the one hand, urbanisation and spatial transformation are driven by economic growth and technical development [20], which can contribute to positive effects on life satisfaction [21]. On the other hand, although economy in cities is fast growing, often urbanisation brings out a decrease in subjective well-being and life satisfaction [22, 23]. This indicates that living in village appears to have a higher level of life satisfaction than living in cities. Indeed, both opposite opinions seems to ignore spatial transformation and personal adjustments that might place direct or indirect impacts on life satisfaction. To address this, Lenzi and Perucca [24] further suggested that there should be higher life satisfaction when rural-to-urban migrants are able to take advantage of economic, social and spatial distributions of urbanisation. Therefore, it is crucial to examine the interaction between characteristics of rural-to-urban transformation and life satisfaction.

However, very few researches have focused on this, there is an urgent need to evaluate migrants’ life satisfaction under urbanisation and examine its influence factors [25, 26]. This study aimed to fill this gap by conducting a survey towards migrants in 18 communities across two major cities of Yangtza Delta in China, seeking to address the central research questions: 1) whether migrants are satisfied with life following urbanisation; 2) which key factors have significantly affected their life satisfaction; 3) how to increase levels of life satisfaction among rural-to-urban migrants.

## Methods

### Research Design

This study adopted a quantitative approach to examine rural-to-urban migrants’ life satisfaction and its key influences. It chose to focus on urbanisation and migration at XS district, Hangzhou City and DJ district, Ningbo City, in Zhejiang province. The reason for this choice is twofold. Firstly, Hangzhou and Ningbo have been selected as pilot cities for implementing national urbanisation policies since 1998, they managed the rural-to-urban migration well. Secondly, XS district and DJ district have developed both monetary compensation and resettlement policies in response to opportunities and challenges brought by integration, the whole urbanisation process has been completed before 2006. In XS district, there are a total of 18 communities, 12 of them were reconstructed to be village-turned-communities, while the rest were newly-built resettlement communities. DJ district used to have 11 villages, but 9 of them have been reconstructed and incorporated into other districts, leaving two village-turned-communities, NF and CB. Therefore, a questionnaire survey was carried out towards migrants in XS and DJ districts. Migrants aged 15 to 64 were included, as they were new urbanites who have experienced transformation in many aspects, including occupational performance, living environment and social identity, which might affect their life satisfaction directly or indirectly.

### Data Collection

Following a review of journal articles and research reports, the questionnaire survey was designed to investigate factors that are associated with new urbanites’ life satisfaction. It was composed of 30 questions in 3 sections, covering basic information, job involvement, willingness and attitude. Prior to the main survey, a pilot study was conducted with 56 migrants in XS district to ensure all survey questions understandable and acceptable. As a result of the pilot, modifications were made to some questions. In order to maximise the response rate and data quality [27], a face-to-face survey was employed and the sample was drawn from the population who shifted from the rural to urban settings and registered in the new urbanised communities between 1998 and 2006. Because communities in XS district are adjacent to each other, rural-to-urban migrants were invited in turn to the service centres of two communities, XY and ZS, for answering the questionnaire. In-house implementation of the survey is feasible and viable when the sample is limited to certain population in specific region [28].

The finalised questionnaire was administered face-to-face in 9 times at 4 community centres. In the end, a total of 877 migrants responded to the survey, 816 were valid to the exclusion of incomplete and inconsistent responses, with a response rate of 93%. As shown in Table 1, 55.02% of all valid responses were collected from XS district in Hangzhou city, while the resting of 44.97% were received at DJ district in Ningbo city. A global measure of life satisfaction (dependent variable) was used as “In general, how satisfied are you with your life after urbanisation?” The answers range from 1 “very satisfied” to 4 “very dissatisfied”? This measure has been continuously adopted by previous studies, such as Chen et al. [29] and Jones [30].

**TABLE 1:** Different levels of response rates across Hangzhou and Ningbo. Study of life satisfaction under urbanisation, China, 2018.

Districts in cities	Communities	N	%
XS in Hangzhou	XY	215	26.34
	ZS	234	28.68
DJ in Ningbo	NF	190	23.25
	CB	177	21.69

### Data Analysis

Survey responses were coded and tested by using SPSS to ensure the samples’ validity and reliability. At the end of the test, Cronbach’s alpha coefficient and KMO value were 0.755 and 0.764 respectively, x^2^ value of Bartlett’s spherical test was 1788.021 (*p* < 0.001), which indicated that the samples were relatively valid and accurate. Descriptive statistics were carried out to reflect a range of factors (independent variables) that may affect migrants’ life satisfaction. These factors were divided into two groups: one described characteristics of social demography, the other explained urbanisation features consisting of occupational change, spatial transformation and identity conversion. Characteristics of social demography were measured by gender, age, educational levels, rural-to-urban years and household wealth. Changes of occupation were linked to career types, specific workplaces, professional skills and job satisfaction. Spatial transformation refers to resettlement and relocation, including community types, housing sectors and community satisfaction. Rural-to-urban identity implies official re-registration of rural villagers as urban residents, which was associated with life adaptation, urban identity, social connection and identity satisfaction.

A variety of methods have been applied to measure the influences of overall satisfaction in different fields, among these logistic regression analysis is the most common tool used in previous research (e.g., [31, 32]). Binary logistic regression is normally used, when the response variable has two possible values, to measure interrelationships between the response variable and potential explanatory variables [33, 34]. It, therefore, was employed in this study.

Since the independent variables are all dichotomous, a binary logistic model was employed for logistic regression analysis. The empirical model was showed as follows:

$$\ln\left(\frac{p_{i}}{1-p_{i}}\right)=\alpha_{0}+\alpha_{1}X_{ij}+\beta Z_{ij}$$

where p_i_ denotes the probability of life satisfaction among the *i*th rural-to-urban migrants; X_ij_ denotes the independent variables of occupational change, spatial transformation and identity conversion; Z_ij_ denotes the control variables of social demography; and β is the coefficient matrix. As the research is aimed at investigating factors influencing life satisfaction from perspectives of socio-demographic and urbanisation features, the explanatory variables were classified into two groups as well as taken into account as a whole to detect their associations with the response variable respectively. Consequently, three binary logistic regression models were performed to determine the relationships between dependent variable and independent variables [35]. First, regression model 1 (RM1) incorporated the first group of socio-demographic characteristics to analyse their impacts on life satisfaction. Then, regression model 2 (RM2) was run to test correlations between the independent variables in the second group of urbanisation features and migrants’ life satisfaction. After that, a holistic model of regression model 3 (RM3) was constructed to identify potential predictors of life satisfaction in both groups. The x^2^ tests of overall significance in regression RM1, RM2 and RM3 were 44.84, 88.46 and 108.83, with the *p* value reaching the significance level of 0.001. This indicates that all regression models provide a better fit and the independent variables are valuable to explain the influences of the overall life satisfaction.

## Results

Table 2 presents descriptive statistics of responses to the dependent variable of overall life satisfaction among rural-to-urban migrants. Because the total responses to “very satisfied” and “very dissatisfied” were merely 6.37% and 1.84%, all answers were divided into two categories for further estimation: I represented “satisfaction” and “very satisfaction”, while II referred to “dissatisfaction” and “very dissatisfaction”. On the whole, 60.78% of migrants reported their life following urbanisation were either satisfactory (54.41%) or very satisfactory (6.37%), while the rest of 39.22% were dissatisfied (37.38%) or very dissatisfied (1.84%) with their current life. This is comparable to other studies, such as Wang [36] and Song [37]. In general, migrants were found greater satisfaction than dissatisfaction with their lives in each city. Hangzhou had the lowest level of life satisfaction at 53.23%, compared with 70.03% in Ningbo and 60.78% in total.

**TABLE 2:** Descriptive statistics of migrants’ life satisfaction across Hangzhou and Ningbo. Study of life satisfaction under urbanisation, China, 2018.

Life satisfaction after urbanisation	Hangzhou (%)	Ningbo (%)	Total (%)	Classification
Very satisfied	4.68	8.45	6.37	I
Satisfied	48.55	61.58	54.41	
Dissatisfied	46.10	26.70	37.38	II
Very dissatisfied	0.67	3.16	1.84	

Table 3 shows survey results associated with independent variables of factors that indicate possible effects on rural-to-urban migrants’ life satisfaction. Based on the nature of each independent variable, these indicators were categorized into two groups: socio-demographic characteristics and urbanisation features. The first group of socio-demographic characteristics was reflected by 5 indicators, consisting of gender, age, education level, rural-to-urban years and household income. More specifically, 50.25% of respondents were male in comparison with 49.75% of female. In China, retirement regimes for workers in different jobs vary, with 60 for men, 55 for women with white-collar jobs and 50 for women with blue-collar jobs. Due to 75.12% of migrants being blue-collar workers after urbanisation, the survey participants were divided into three age groups, covering 26.23% of young adulthood aged 18 to 35, 37.38% of middle age from 36 to 50 and 36.40% of older adults aged 51 or over. Except for 23.90% receiving college or university degrees, the remaining participants were educated in lower levels (high school or below). They all have been officially registered in a urbanised community for 11 years or over, with 37.87% being urbanities for more than 13 years. Despite a wide variety of net annual household income, on average most families earned less than 60k in a year.

**TABLE 3:** Summary statistics of independent variables in relation to social demography, occupational change, spatial transformation and identity conversion. Study of life satisfaction under urbanisation, China, 2018.

Independent variables	Coding	%	Mean	Std.dev	Min	Max	Characteristics
Gender	0 = Female	49.75	0.5025	0.5003	0	1	Social demography
	1 = Male	50.25					
Age	1 = Under 35 years	26.23	2.1017	0.7853	1	3	
	2 = 36 to 50	37.38					
	3 = 51 years and over	36.40					
Level of education	1 = Illiteracy	3.55	3.3836	1.1707	1	5	
	2 = Primary school	22.06					
	3 = Secondary school	30.76					
	4 = High school	19.73					
	5 = College and over	23.90					
Rural-to-urban years	0 = Less than 13 years	62.13	0.3787	0.4854	0	1	
	1 = 13 years and over	37.87					
Net annual household income (10k RMB)	Continuous		5.80	5.42			
Categories of occupation	1 = Unemployed	7.23	2.8493	0.8781	1	4	Occupational change
	2 = Flexible	25.49					
	3 = Physical	42.40					
	4 = Managerial or Technical	24.88					
Types of workplaces	1 = Unemployed	7.23	2.7708	0.9465	1	5	
	2 = Individual business	32.48					
	3 = Private company	40.93					
	4 = State owed enterprise	14.71					
	5 = State Organs and public institution	4.66					
Professional skill	0 = No	84.19	0.1581	0.3651	0	1	
	1 = Yes	15.81					
Satisfaction with current job	1 = Poor	0.86	2.4081	0.5090	1	3	
	2 = Moderate	57.48					
	3 = High	41.67					
Types of community	0 = Village-turned-ommunity	21.69	0.7831	0.4124	0	1	Spatial transformation
	1 = Resettlement community	78.31					
Types of housing	0 = Self-built or rental	28.60	0.7525	0.4319	0	1	
	1 = Resettled	71.40					
Satisfaction with community environment	1 = Poor	0.60	2.5147	0.5122	1	3	
	2 = Moderate	47.30					
	3 = High	52.08					
Adaptability to city life	1 = Poor	8.21	2.1324	0.5286	1	3	Identity conversion
	2 = Fair	70.34					
	3 = Good	21.45					
Identification with urban citizenship	1 = Poor	15.93	2.0417	0.5992	1	3	
	2 = Fair	63.97					
	3 = Good	20.10					
Social connections	1 = Poor	56.00	1.6789	0.8347	1	3	
	2 = Fair	20.10					
	3 = Good	23.90					
Satisfaction with identity transformation	1 = Poor	0.86	2.4755	0.5166	1	3	
	2 = Moderate	50.74					
	3 = High	48.4					

The second group of urbanisation features spans three aspects of occupational change, spatial transformation and identity conversion, with a total of 11 indicators. Urbanisation involves the transition of the rural to urban society that has resulted in some changes to migrants’ occupation. The statistical results disclosed that 75.12% of migrants were blue-collar workers in carrying out physical or flexible jobs compared with 24.88% working as white-collar (e.g., managers, technicians). Likewise, 84.19% of migrants were not qualified with professional skills; they were mainly employed by private enterprises (40.93%) or individual business (32.48%). There were still 7.23% being out of employment. The overall job satisfaction was good, with 41.67% ranking high satisfaction with the current job.

Besides, spatial evaluation of developing communities and living environments occurs with urbanisation. Often urbanisation brought about two typical communities of the resettlement community and the village-turned-community, with the former taking up 78.31% and the latter accounting for the rest. Following transformation from villages to cities, 71.40% of migrants moved into resettled houses, only 28.60% built their own homes or rented the properties. Almost all migrants were either moderately (47.30%) or highly (52.08%) satisfied with their community environments. In addition, urbanisation involves the flow of rural residents into newly constructed cities, which is linked to some changes towards their daily lifestyles, urban identities and social connections. Although there were many differences between rural and urban lifestyles, 91.79% of migrants found their ability to accommodate the city life either fair (70.34%) or good (21.45%). Their recognition of the new urban identity, likewise, was considerably high at 84.07%. However, communication networks have been seriously disrupted, over half migrants claimed to have a lower social interaction in cities than that in villages. Despite this, the total satisfaction with different dimensions of urban identity transformation were found fairly high.

Table 4 displays three logistic binary regression models of RM1, RM2 and RM3 that highlight correlations between dependent variable and independent variables. Specifically, RM1 includes only variables related to socio-demographic characteristics of the respondents, in order to examine their associations with life satisfaction. It shows that age, education, and net annual household income are significant predictors of life satisfaction. There was a negative correlation between the age group of 36–50 and life satisfaction. Relationships between education levels and life satisfaction were significant, which means migrants with better education were found to have higher life satisfaction. Also, the net annual household income had a positive effect on life satisfaction with the significance level of *p* < 0.001. Other variables, such as gender and rural-to-urban years, were found no correlation with life satisfaction.

**TABLE 4:** Results of logistic meta-analysis about the influences on life satisfaction. Study of life satisfaction under urbanisation, China, 2018.

Independent variables	RM1		RM2		RM3	
	B	Exp(B)	B	Exp(B)	B	Exp(B)
Gender (Female)	0.212	1.236			0.146	1.157
Age (Under 35 years)						
36–50	−0.641*	0.527			−0.128	0.880
≥51	−0.157	0.854			−0.205	0.815
Level of education (College and over)						
Illiteracy	−2.325**	0.098			−2.127**	0.119
Primary school	−0.733**	0.481			−0.745*	0.475
Secondary school	−0.666*	0.514			−0.684*	0.504
High School	−0.540*	0.583			−0.506*	0.603
Rural-to-urban years (Less than 13 years)	−0.203	0.816			−0.083	0.920
Net annual household income (10k RMB)	0.052**	1.053			0.044**	1.045
Categories of occupation (Managerial and technical)						
Unemployed			−0.406	0.006	−0.593	0.098
Flexible			−0.260	0.297	−0.327	0.386
Physical			−0.148	0.244	−0.226	0.436
Professional skill (No)			0.124	1.132	0.071	1.074
Types of workplaces (State Organs and public institution)						
Unemployed			−1.286*	0.276	−1.137**	0.321
Individual work			−0.798*	0.450	−0.767*	0.513
Private enterprise			−0.499	0.607	−0.428	0.652
State owed enterprise			−0.241	0.786	−0.080	0.923
Satisfaction with current job (High)						
Poor			−0.269	0.512	−0.257	0.245
Moderate			−0. 189	0.764	−0.108	0.773
Types of community (Village-turned-community)			0.756**	1.927	0.889**	1.416
Types of housing (Self-built or rental)			0.312	1.366	0.736	1.087
Satisfaction with community environment (High)						
Poor			−0.201	0.601	−0.104	0.541
Moderate			−0.084	0.919	−0.028	0.792
Social connections (Good)						
Poor			−0.064	0.066	−0.052	0.054
Fair			−0.162	0. 176	−0.452	0.572
Identification with urban citizenship (Good)						
Poor			−0.845*	0.430	−0.647	0.524
Fair			−0.434*	0.791	−0.182	0.827
Adaptability to city life (Good)						
Poor			−1.448**	0.235	−1.417**	0.243
Fair			−0.706**	0.493	−0.739**	0.478
Satisfaction with identity transformation (High)						
Poor			−0.164	0. 179	−0. 139	0. 149
Moderate			−0.129	0. 879	−0. 323	0. 724
Constant	1.231	3.425**	0.406**	1.501	0.384	1.468
- 2Loglikelihood	106.14		100.45		118.41	

Note: Reference groups in parentheses, *p < 0.05, **p < 0.01, ***p < 0.001.

RM2 inputs only variables of occupational change, spatial transformation and identity conversion in the second group to estimate correlations between urbanisation features and life satisfaction. It found that workplaces, community types, urban citizenship, adaptability had direct influences on life satisfaction. Relationships between workplace types and life satisfaction varied substantially, ranging from no correlation to negative correlation. In other words, migrants who worked in the private company or state owned enterprise had higher levels of life satisfaction than those running own business or being unemployed. Following demolition of villages, rural residents were arranged to settle either in a newly-built resettlement community or a village-turned-community. The community types can affect life satisfaction, rural villagers who relocated in the resettlement community were more satisfied with their life than those remained in the village-turned-community. Similarly, identification of urban citizenship has positive effect on life satisfaction, migrants with a stronger sense of urban citizenship tended to have greater life satisfaction. Adaptability to the city life was associated with life satisfaction, new urbanites who adapted well to life in urban environments reported a higher life satisfaction than others. The remaining indicators, including occupational categories, qualifications, job satisfaction, housing types, community satisfaction and social connection, were measured to have no correlations with life satisfaction.

RM3 brings in all variables of both the first and second groups to measure their impacts on life satisfaction. Obviously, there are certain changes with significant effects of the same variables between different models. This is because the regression coefficients normally depend on variables that are included in a model, they will change their value (e.g., from significant to non-significant) when other variables are added into the model [38]. As a whole, education, income, workplace, community and adaptability were significantly linked to life satisfaction. Levels of life satisfaction generally came down along with the fall in education levels. Positive correlation between net annual household income and life satisfaction was found, the raise of every 10k in family income indicates that the level of life satisfaction goes up by 4.5%. Types of workplaces had direct effects on life satisfaction, self-employed or unemployed migrants reported a relatively lower life satisfaction. Rural villagers relocated in the resettlement community were more satisfied with their life compared with those lived in the village-turned-community. Meanwhile, the levels of life satisfaction fluctuated with personal adaptability to the city life, migrants who have been well adapted to urban life had a greater likelihood of being satisfied with their life. Other variables in the first and second groups were found no correlations with life satisfaction.

## Discussion

In spite of a relatively high life satisfaction, there were still over one third of rural-to-urban migrants reporting unsatisfied with their current life. Four essential drivers are found to have direct influences on migrants’ life satisfaction: social demography, occupational change, spatial transformation and identity conversion. From the perspective of socio-demographic characteristics, the majority of migrants were older than 35 years, received high school education or below, became new urbanites for less than 13 years, and had an average net annual household income under 60k. Among these characteristics, education and income were strongly related to life satisfaction. The variable of age was a very weak predictor of life satisfaction, the detrimental effect of the age group 36–50 on life satisfaction was reduced from significant to non-significant in the full model. In particular, relationships between age groups and life satisfaction originally displayed a U shape, life satisfaction reached the bottom at the age group 36–50. This indicates that migrants aged 36-50 are likely to be less satisfied with their life than those in age groups ≤35 and ≥51, which is in line with the findings from Song’ study [37]. One inference from it could be, migrants in their middle age take on various roles of pursuing career development, caring for family and performing other duties that might affect their satisfaction with life, while those aged older than 50 are mainly approaching retirement or in retirement and they are less pressured by social responsibilities [39]. Despite the potential influence of the age group 36–50, the value of age was weakened by other significant influences, leaving insignificant effects on overall life satisfaction. In general, educational attainment plays an important role in improving career choices, identity conversion and environment adaptiveness [40, 41], which is linked to subjective well-being and life satisfaction. Unsurprisingly, migrants with higher education reported to have higher levels of life satisfaction than others. In addition, education has been recognized as one core determinant of the income level [42], and economic stability can guarantee the fulfillment of basic needs as well as the enhancement of personal well-being [36]. This was also evidenced in this study that household income had a direct impact on migrants’ life satisfaction.

In terms of these characteristics of social demography, overall, migrants with higher education and earnings were more likely to make use of urban externalities and to have greater life satisfaction. As evidenced in previous studies [43, 44], education achievement enables individuals to take advantage of existing opportunities and resources as well as make a quick adaptation to the new environment, leading to better personal well-being and life satisfaction. Therefore, it is of most importance for policy officers to give increased emphasis on educational attainment for rural-to-urban migrants, especially those in the young age group. Given the positive association between income distribution and life satisfaction, there should be policies and actions in place to maximise the family’s financial resources and annual income. Except for economic compensation from the government, it is essential to develop the collective economy of rural-to-urban communities, stimulate the creation of local jobs and reduce the household’s reliance on the rental housing, in order to ensure migrants’ access to employment stability and income security.

Urbanisation inevitably involves occupational change, spatial transformation and identity conversion for migrants. With respect to the nature of occupation, new urbanites were basically engaged in physical or flexible work, employed by either themselves or private enterprises and lack of professional skills, but had a relatively high satisfaction with their current work. Of theses factors, the types of workplaces were evaluated to significantly affect life satisfaction, as reported in Weziak-Bialowolska’s research [45]. Employment in formal sectors or informal sectors is expected to have a direct or indirect effect on the level of life satisfaction [46]. Similarity in this study, formal sectors (e.g., public institutions, state owned enterprises and private companies) were associated with higher life satisfaction compared with informal sectors (e.g., individual business). There could be several potential reasons [25, 47, 48]: first, types of workplaces are closely connected with personal development and career success, formal sectors in China have tended to create more opportunities for improvement of professional skills and living standards; second, formal contractual arrangements can help workers entry into stable employment and high-quality life; third, organisations like public institutions and state owned enterprises normally provide a range of welfare benefits and social services for their employees. Consequently, public institutions, private enterprises and the third sector should work in partnership to increase the pool of rural-to-urban migrants in formal sectors. This requires equal attention to the development of private industry and collective economy in rural-to-urban areas, for the purpose of opening up more formal jobs and improving the employment outlook for migrant workers. Meanwhile, to increase migrants’ labor market participation, projects should be designed to facilitate migrants’ access to skill development, professional training and vocational education. Since welfare benefits are largely subject to workplaces, job satisfaction was found no correlation with life satisfaction.

Typically there is spatial transformation from rural to urban environments under urbanisation [49]. In this study, most of migrants moved into resettled houses in the resettlement community and felt satisfied with the community environment. More specifically, life satisfaction in the resettlement community was higher than that in the village-turned-community. Compared to the village-turned-community, the resettlement community is newly built by local government with unified planning, affiliated facilities and inhabited environment [50, 51]. It has greater potential to promote friently living environment that is viewed as the affective component of life satisfaction [52]. By contrast, fundamental changes are required to enhance the quality of urban design and environment in the village-turned-community. In order to achieve sustainable urban development, on average migrants are offered a compensation package by the government including cash payment and several apartments for their relocation. With the exception of one owner occupied, other apartments are often rented out for extra income, which sometimes is higher than individual salary and becomes the primary economic resource for the household. Higher income levels can contribute to a boost in life satisfaction and well-being [26]. This, however, brought a range of managerial challenges for local communities due to frequent movements of tenants. To address it, there is a need for local government to take advantage of innovations for service provision and performance management. Meanwhile, it is necessary to raise awareness of the relationship between community environment and quality of life. Both property types and community environment was found no association with life satisfaction, indicating that migrants have paid more attention to rental payments instead of property quality or community wellness.

Since urbanisation brings about an identity change and a strange surroundings [44], migrants have made certain adjustments to urban identity and city life There were few social interactions among migrants, but they were generally satisfied with their identity transformation. Similar to the variable of age, the significant effect of identification with urban citizenship on life satisfaction decreased to be insignificant when other variables had a higher value. In other words, transformation of urban identity was positively linked to life satisfaction, but its influence was relatively weak. Comparatively, life satisfaction run higher when migrants were capable of making certain changes to the city life. Following urbanisation and migration, rural villagers have to experience socio-cultural transition, including social integration and adaptation [53]. Such transition is a graduate process, which requires self-efficacy and empowerment. Self-efficacy refers to build up personal strength and capability to act in a new situation and achieve an expected outcome [54], while empowerment mainly focus on external support to individual’s rights, needs and choices that maximise their participation in social changes and daily activities [55]. Therefore, on the one hand, rural-to-urban migrants should take active steps to adjust to their new community, develop a sense of belonging and get involved in community engagement. On the other hand, local government should take a variety of positive actions, such as improving understanding of migrants’ demands, increasing access to community services, expanding labour supply and creating social participation opportunities. These actions can help migrants cope with occupational, social and cultural changes and adapt to the new surroundings, and further to achieve a higher level of life satisfaction.

### Limitations

While this study brought new understanding of factors influencing migrants’ life satisfaction, there are some limitations. First, this study selected samples from rural-to-urban communities across developed regions in China, it did not investigate same types of communities in less developed regions. Therefore, caution should be taken when generalising the predictors of life satisfaction across all urbanised areas. Besides, the research focused mainly on factors affecting migrants’ life satisfaction following socio-spatial transformation, with little attention paid to the influences at the community level and to more opportunities for children after urbanisation. Further research is needed to identify the community elements and children’s development that might have direct effects on the migrants’ life satisfaction, so that rural-to-urban migration can remain stable, motivated and modernised.

### Conclusion

China has seen a rapid urbanisation with the expansion of migration from rural villages to urban cities [56]. New urbanites are faced with a variety of social changes and challenges that might have positive or negative effects on their life satisfaction. Both national and local policies have made a commitment to improve life satisfaction and well-being among rural-to-urban migrants. However, the current implementation of these policies is limited, the overall life satisfaction under urbanisation has not proved as high as expected, with over one third of migrants reporting dissatisfied with their life. This was influenced by factors relating to social demography and urbanisation features. Socio-demographic characteristics like educational level and net annual household income were significantly linked to life satisfaction. More specifically, when migrants received a higher education or income, they tended to have a greater life satisfaction. This reflects economic determinant of life satisfaction as well as an requirement to improve migrants’ educational levels and financial resources.

Urbanisation is normally accompanied by occupation change, spatial transformation and identity conversion. Migrants worked in formal sectors (e.g., public institutions and state owned enterprises) were more satisfied with their life than those in informal sectors or unemployment. Positive actions, such as providing professional training and developing vocational skills, can be taken to maximise labor participation in formal sectors. Rural villagers, who moved into the newly-built resettlement community, appeared to have higher life satisfaction than those who remained in the village-turned-community. There should be fundamental changes to physical, social and economic environment in the village-turned-community. Likewise, making an adaptation to urban life can stimulate a sense of community belonging, leading to a higher level of life satisfaction. Therefore, there is a need not only for migrants to build up personal power and strength but also for local government to guarantee welfare benefits and social interactions.
